# Using surveillance data to inform a SUID reduction strategy in Massachusetts

**DOI:** 10.1186/2197-1714-1-12

**Published:** 2014-05-06

**Authors:** Nicole J Treadway, Hafsatou Diop, Emily Lu, Kerrie Nelson, Holly Hackman, Jonathan Howland

**Affiliations:** 1 Boston Medical Center Injury Prevention Center, Boston, MA USA; 2 Department of Emergency Medicine, Boston University School of Medicine, One Boston Medical Center Place, Dowling 1, South, Boston, MA 02118 USA; 3 Massachusetts Department of Public Health, 250 Washington Street, 02108 Boston, Massachusetts USA; 4 Boston University School of Public Health, 715 Albany Street, Talbot Building, 02118 Boston, MA USA

**Keywords:** SUID, SIDS, Infant sleep, PRAMS, Supine sleep, WIC, Data to action

## Abstract

**Background:**

Non-supine infant sleep positions put infants at risk for sudden unexpected infant death (SUID). Disparities in safe sleep practices are associated with maternal income and race/ethnicity. The Special Supplemental Nutrition Program for Women, Infants and Children (WIC) is a nutrition supplement program for low-income (≤185% Federal Poverty Level) pregnant and postpartum women. Currently in Massachusetts, approximately 40% of pregnant/postpartum women are WIC clients. To inform the development of a SUID intervention strategy, the Massachusetts Department of Public Health (MDPH) investigated the association between WIC status and infant safe sleep practices among postpartum Massachusetts mothers using data from the Pregnancy Risk Assessment Monitoring System (PRAMS) survey.

**Methods:**

PRAMS is an ongoing statewide health surveillance system of new mothers conducted by the MDPH in collaboration with the Centers for Disease Control and Prevention (CDC). PRAMS includes questions about infant sleep position and mothers’ prenatal WIC status. Risk Ratio (RR) and 95 percent confidence intervals (CI) were calculated for infant supine sleep positioning by WIC enrollment, yearly and in aggregate (2007–2010).

**Results/Outcomes:**

The aggregate (2007–2010) weighted sample included 276,252 women (weighted n ≈ 69,063 women/year; mean survey response rate 69%). Compared to non-WIC mothers, WIC mothers were less likely to usually or always place their infants in supine sleeping positions [RR = 0.81 (95% CI: 0.80, 0.81)]. Overall, significant differences were found for each year (2007, 2008, 2009, 2010), and in aggregate (2007–2010) by WIC status.

**Conclusion:**

Massachusetts WIC mothers more frequently placed their babies in non-supine positions than non-WIC mothers. While this relationship likely reflects the demographic factors associated with safe sleep practices (e.g., maternal income and race/ethnicity), the finding informed the deployment of an intervention strategy for SUID prevention. Given WIC’s statewide infrastructure and the large proportion of pregnant/postpartum women in Massachusetts that are enrolled in WIC, a WIC-based safe sleep intervention may be an effective SUID reduction strategy with potential national application.

**Electronic supplementary material:**

The online version of this article (doi:10.1186/2197-1714-1-12) contains supplementary material, which is available to authorized users.

## Background

Sudden Unexpected Infant Death (SUID) includes explained and unexplained infant deaths. Sudden Infant Death Syndrome (SIDS) is the unexplained death of infants less than one year of age (CDC (2012b)). Sleep environment and positioning can be a cause of SUID and a possible (though undetected) cause of SIDS due to asphyxiation, suffocation, or entrapment (Moon & Fu, 2012). The prone (stomach) sleep position is associated with an increased risk of infant death (Gilbert et al. 2005). In 1992, the American Academy of Pediatrics (AAP) recommended that infants be placed to sleep in a non-prone (back or side) position. This recommendation, along with the “Back to Sleep” campaign launched by the National Institute of Child Health and Human Development in 1994, was credited with lowering the rate of SIDS between 1992 and 2001 by 53% (Task Force on Sudden Infant Death Syndrome, 2011). Thereafter, the rate of SIDS leveled off, remaining constant from 2001 to 2006 (Task Force on Sudden Infant Death Syndrome, 2011). The National Infant Sleep Position (NISP) study indicates that between 2001 and 2007, the number of infants placed to sleep in the supine (back) position plateaued as well (Colson et al., 2009).

In 2005, the AAP modified its recommendations stating that infants be placed for sleep in the supine position *only*. This was reiterated by the AAP in 2011 along with recommendations addressing sleep location and environment (Task Force on Sudden Infant Death Syndrome, 2011). Preventive efforts targeting safe sleep continue because recommended infant sleep practices are not universally adopted. One of the objectives of the Healthy People 2020 is to increase the proportion of infants placed on their backs to sleep from 69.0% in 2007, to 75.9% by 2020 (Maternal, Infant, and Child Health - Healthy People 2012).

Supine infant sleep position is associated with maternal and infant demographics (Colson et al., 2009). Studies consistently indicate that black infants are less likely than white infants to be placed in the supine position (Hauck et al., 2002; Lesko et al., 1998; Pollack & Frohna, 2001; Willinger et al. 2000). NISP data since 2001 show that the proportion of white infants being put to sleep in the supine position remained constant at 75% while that for infants of black women remained constant at 58% (Colson et al., 2009). Greater rates of SIDS and non-supine sleep have also been linked to low income (Corwin et al., 2003; Pickett et al. 2005). Thus, achieving the Healthy People 2020 objective for infant safe sleep will require reducing socio-demographic disparities in safe sleep practices.

The Special Supplemental Nutrition Program for Women, Infants and Children (WIC) is a federally funded program providing supplemental foods, health care referrals, nutrition education, and breastfeeding support to low-income pregnant and postpartum women, and children up to five years (WIC’s Mission 2012). In 2010, of the approximately 10 million women and children that were enrolled in WIC nationally, 70% were at or below the Federal Poverty Level (FPL) (Connor et al. 2011). Approximately 40% of all women giving birth in Massachusetts in 2009 reported that they were enrolled in the WIC program (Massachusetts Department of Public Health, 2012) and according to the 2011 Massachusetts (MA) Pregnancy Nutrition Surveillance System, 29.0% of MA women served by WIC are at or below 50% of the FPL (Massachusetts Department of Public Health and Bureau of Family Health and Nutrition 2011). In MA, among women served by WIC, 56.8% are non-white, including 17.4% that identify as black, non-Hispanic and 32.5% that identify as Hispanic (Massachusetts Department of Public Health and Bureau of Family Health and Nutrition 2011). Over 5,000 black infants and nearly 9,000 Hispanic infants were served in MA in 2010 (Connor et al., 2011).

Pursuant to funding for the Core Violence and Injury Prevention Program (VIPP) from the Centers for Disease Control and Prevention (CDC), the MA Department of Public Health (MDPH) developed a strategic plan for unintentional injury prevention. One aim is to reduce infant deaths by reducing demographic disparities in safe sleep practices. Overlapping demographics between WIC participants and at-risk populations for unsafe sleep practices (Connor et al., 2011; Massachusetts Department of Public Health and Bureau of Family Health and Nutrition 2011) suggested that MA WIC mothers would be less likely to place their babies in the supine position than non-WIC mothers. To investigate this, we analyzed recent Pregnancy Risk Assessment Monitoring System (PRAMS) surveillance data of new MA mothers. This paper describes the association between maternal WIC enrollment and reported infant sleep practices. The aim is not to explain why WIC mothers are less apt than non-WIC mothers to place their infants in safe sleep positions; rather, our purpose is: 1) To generally illustrate how surveillance data that is routinely collected for public purposes can be used to inform the development of intervention strategies; and 2) To specifically inform the deployment of a SUID risk reduction strategy. The identified association between maternal WIC participation and reported infant sleep practice, as well as the statewide network that WIC provides, led the MDPH Injury Prevention and Control Program to partner with the MA WIC program and the Children’s Safety Network (CSN) to develop and implement a statewide safe sleep intervention.

## Methods

### Survey methods

We used MA PRAMS data as the primary data source for our analysis. PRAMS is an ongoing statewide surveillance system managed by the MDPH in collaboration with the CDC. PRAMS annually surveys new MA mothers about their experiences and behaviors before, during and after pregnancy. It is administered in English and Spanish (Massachusetts Department of Public Health, 2012). Participants are randomly selected (2–6 months postpartum) from in-state birth certificates of all live-born infants among MA-resident mothers (excluding multiples >3, adopted infants and surrogate births). To ensure adequate representation of minority populations, MA PRAMS over-samples by race and Hispanic ethnicity. Data are weighted to account for the complex survey design, non-coverage, and non-response (CDC 2012a: website: http://www.cdc.gov/prams/Methodology.htm). Data from 2007 through 2010 were analyzed independently by year and in aggregate. The weighted aggregate PRAMS sample included 276,252 women, ranging between 67,140 and 70,782 each year, over the four years. Participants were mailed up to three paper surveys, after which mail non-respondents were surveyed by telephone. Survey response rates averaged 69% (Massachusetts Department of Public Health, 2012). The MA PRAMS study is approved by the MDPH Institutional Review Board.

### Measurement

To assess safe sleep positioning respondents were asked “In which *one* position do you *most often* lay your baby down to sleep now?” Exclusive response options were: “On his or her side”, “On his or her back” or “On his or her stomach”. Side sleep and stomach sleep were combined as “non-supine sleep.” The independent variable was assessed by the question, “During *your most recent* pregnancy, were you on WIC (the Special Supplemental Nutrition Program for Women, Infants, and Children)?”

### Analysis

Chi-square tests were used to examine differences in socio-demographic characteristics of the mothers by WIC status. Risk ratio (RR) and 95 percent confidence intervals (CI) were calculated for infant supine sleep position by WIC status, yearly and in aggregate (2007–2010) using logistic regression models. When comparing prevalence estimates across sub-groups, p-value was considered statistically significant at alpha < 0.05. We used SAS version 9.2 (SAS Institute Inc., Cary, NC) to clean and recode PRAMS data, and SUDAAN version 10.0 (Research Triangle Institute, Research Triangle Park, NC) to analyze and generate weighted prevalence estimates and RR point estimates with 95% CI. Chi-square p-value was calculated using Wald-F testing in SUDAAN.

## Results

### Demographics in aggregate sample

WIC mothers and non-WIC mothers were significantly different with regard to race/Hispanic ethnicity, marital status, maternal age, education, household income per household size by federal poverty level, and preferred language (Table 1).

**Table 1:** **Differences in demographics between WIC and non-WIC mothers of infants, 2007–2010, MA PRAMS, USA**

Category	Demographic	% WIC mothers (n ^w^ = 102,948)	% Non-WIC mothers (n ^w^ = 173,304)	Chi-square P value
Marital status	Married	31.8	84.8	<0.001
	Single	68.2	15.2	
Maternal age (years)	<20	13.6	1.6	<0.001
	20-29	59.5	31.2	
	30-39	25.0	62.0	
	40+	1.9	5.2	
Maternal education	< High School	22.5	2.5	<0.001
	≥ High School	77.5	97.5	
Household Income- Federal Poverty Level (FPL)	> 100% FPL	47.5	94.5	<0.001
	≤ 100% FPL	52.5	5.5	
Preferred language	English preferred	78.2	96.3	<0.001
Parity	No previous births	47.3	50.3	0.0997
	Yes previous Births	52.7	49.7	
Maternal race/Ethnicity	White, non Hispanic	44.9	81.5	<0.001
	Black, non Hispanic	16.4	3.9	
	Hispanic	30.3	4.9	
	Asian, non Hispanic	6.3	8.9	
	Other	2.1	0.7	

n^w^: weighted sample size.

### Disparities in supine sleep by WIC status

**Figure 1:**
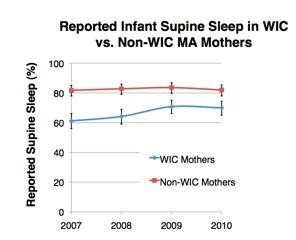
**Percent of WIC (n**^**w**^ **= 102,948) and non-WIC (n**^**w**^ **= 173,304) mothers reporting supine infant sleep, 2007–2010, MA PRAMS.**

In the aggregate analysis (including 2007–2010), the overall prevalence of infants placed to sleep in the supine position was 76.6% in MA. WIC mothers were significantly less likely than non-WIC mothers to report usually placing their infants in the supine sleep position (66.5% vs. 82.5%; RR = 0.81; 95% CI: 0.77-0.84). This finding was significant and consistent for each of the survey years (Figure 1, Table 2).

**Table 2:** **Prevalence and risk ratio of infant supine sleep; WIC vs. non-WIC mothers, 2007–2010, MA PRAMS**

Year	sample size (n ^w^)	WIC mothers (n ^w^)	Non-WIC mothers (weighted (n ^w^)	Risk ratio (95% CI)*	P-value
2007	70,782	26,462	44,320	0.75 (0.68-0.82)	.0001
2008	70,456	25,605	44,851	0.78 (0.71-0.85)	.0001
2009	67,874	26,050	41,824	0.85 (0.79-0.91	.0001
2010	67,140	24,831	42,309	0.85 (0.79-0.93)	.0001
Aggregate (2007–2010)	276,252	102,948	173,304	0.81 (0.77-0.84)	.0001

n^w^: Weighted sample size.

*Reference group: non-WIC mothers.

## Discussion

These data indicate that MA WIC mothers are less likely to place their babies in the safer supine sleep position than non-WIC mothers. Consequently, we were able to identify an access point to educate at-risk populations about safe sleep through the WIC program. In partnership with the CSN and MA WIC program, MDPH is using this access point to disseminate the recommendations put forth in the 2011 AAP infant safe sleep guidelines. MDPH is training WIC supervisors to subsequently train all MA WIC area office staff about safe infant sleep practices, including information on sleep position, sleep surface, and sleep environment. The training also identifies protective factors and addresses common concerns of at-risk populations, including concerns previously identified in the literature about comfort and choking (Colson et al., 2006,2009; Robida & Moon, 2012). The aim of the training is to have WIC counselors promote standardized infant sleep messaging when working with clients during pregnancy and/or in the postpartum period. Intervention evaluation will include monitoring safe-sleep positioning in future MA PRAMS surveys.

Our findings and conclusions are similar to those of the Hawai’i Department of Public Health while our maternal demographics were different from each other (Schempf et al. 2011). Using Hawai’i PRAMS data from 2004–2008, Schempf et al. found that 36.4% of women who participated in prenatal WIC services placed their babies in non-supine positions as compared to 26.8% of women that did not participate in the WIC program. Findings of the Hawai’i and MA Health Departments may be generalizable to other states. If so, intervening for infant safe sleep via the WIC program might inform injury prevention policy nationally.

Our findings, however, are subject to limitations. WIC status and infant sleep position are self-reported in PRAMS and remain unverified. Furthermore, the categorization of women as WIC or non-WIC does not account for duration of enrollment in WIC or for non-WIC mothers that are WIC eligible based on their income level. These factors, however, would tend to diminish differences by WIC status, and therefore differences observed may be underestimated. Finally, PRAMS data do not reveal why WIC mothers are less apt to adopt safe sleep practices and thus the intervention may not fully address barriers to behavioral change. This does not alter, however, the importance of WIC as a potential venue for promoting safe sleep practices. Future evaluation of the present program would determine intervention effectiveness and whether alternative behavioral change strategies are required.

## Conclusion

This investigation illustrates the concept of data-to-action; it provides an example of using surveillance data to inform the development of public health strategies. Analysis of PRAMS data indicated that WIC mothers were at greater risk of sleeping their infants unsafely as compared to non-WIC mothers. These findings identified WIC mothers for targeted intervention to reduce infant mortality and led investigators to determine that the WIC program, with its existing infrastructure, is a potential vehicle for closing the disparity gap in reported infant safe sleep practice. If this strategy for safe sleep education dissemination proves effective in MA, it may provide a model for reducing safe sleep disparities nationally.

## Authors’ original submitted files for images

Below are the links to the authors’ original submitted files for images. Authors’ original file for figure 1

## Abbreviations

AAP: American Academy of Pediatrics
CDC: Centers for Disease Control and Prevention
CSN: Children’s safety network
FPL: Federal Poverty Level
MA: Massachusetts
MDPH: Massachusetts Department of Public Health
NISP: National Infant Sleep Position Study
PRAMS: Pregnancy Risk Assessment Monitoring System
WIC: Special Supplemental Nutrition Program for Women, Infants and Children
SIDS: Sudden Infant Death Syndrome
SUID: Sudden Unexpected Infant Death.
