# Supplementary figures and images for: Using surveillance data to inform a SUID reduction strategy in Massachusetts
Source: Inj Epidemiol. 2014 May 6;1(1):12. doi: 10.1186/2197-1714-1-12 (PMC5005622; doi:10.1186/2197-1714-1-12)

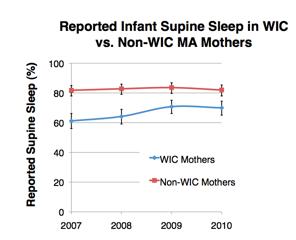

Supplement: Supplementary file 1 — Authors’ original file for figure 1 [file 40621_2013_12_MOESM1_ESM.jpeg]
